# Masitinib antagonizes ATP-binding cassette subfamily G member 2-mediated multidrug resistance

**DOI:** 10.3892/ijo.2014.2341

**Published:** 2014-03-13

**Authors:** RISHIL J. KATHAWALA, JUN-JIANG CHEN, YUN-KAI ZHANG, YI-JUN WANG, ATISH PATEL, DE-SHEN WANG, TANAJI T. TALELE, CHARLES R. ASHBY, ZHE-SHENG CHEN

**Affiliations:** 1 Department of Pharmaceutical Sciences, College of Pharmacy and Health Sciences, St. John’s University, Queens, NY, USA;; 2 Department of Medical Oncology, Sun Yat-sen University Cancer Center; State Key Laboratory of Oncology in South China; Collaborative Innovation Center for Cancer Medicine, Guangdong, P.R. China

**Keywords:** ATP binding cassette subfamily G member 2, tyrosine kinase inihibitors, multi-drug resistance, masitinib

## Abstract

In this *in vitro* study, we determined whether masitinib could reverse multidrug resistance (MDR) in cells overexpressing the ATP binding cassette subfamily G member 2 (ABCG2) transporter. Masitinib (1.25 and 2.5 *μ*M) significantly decreases the resistance to mitoxantrone (MX), SN38 and doxorubicin in HEK293 and H460 cells overexpressing the ABCG2 transporter. In addition, masitinib (2.5 *μ*M) significantly increased the intracellular accumulation of [^3^H]-MX, a substrate for ABCG2, by inhibiting the function of ABCG2 and significantly decreased the efflux of [^3^H]-MX. However, masitinib (2.5 *μ*M) did not significantly alter the expression of the ABCG2 protein. In addition, a docking model suggested that masitinib binds within the transmembrane region of a homology-modeled human ABCG2 transporter. Overall, our *in vitro* findings suggest that masitinib reverses MDR to various anti-neoplastic drugs in HEK293 and H460 cells overexpressing ABCG2 by inhibiting their transport activity as opposed to altering their levels of expression.

## Introduction

One of the major problems associated with the treatment of cancer is the development of resistance to chemotherapeutic drugs (1,2). The most common mechanisms that produce drug resistance in cancer cells include: i) altered cell cycle check points; ii) induction of emergency response genes; iii) alterations in membrane lipids; iv) compartmentalization; v) inhibition of apoptosis; vi) altered drug targets; vii) decreased uptake and viii) increased efflux of drugs (2–6). One type of resistance that is common and highly problematic is multidrug resistance (MDR). MDR occurs when cancer cells become resistant to structurally and mechanistically distinct classes of chemotherapeutic compounds (2,7,8). A common mediator of MDR in cancer cells is the family of specific transmembrane, energy-dependent transporters known as ATP-binding cassette (ABC) transporters (9). The ABC transporter family is divided into seven subfamilies, ABCA through ABCG (10,11). Currently, 48 members of the ABC transporter family have been isolated and identified (1,11). Mechanistically, the catalytic cycle of the transporter involves two ATPs. The first molecule of ATP is hydrolyzed by ATPase, producing a structural modification of the trans-membrane domains that flips the inner membrane leaf to the outside of cell membrane, thereby removing or effluxing the compound. The second ATP is hydrolyzed to restore the transporter back to its original high affinity state for substrate transport (12). The breast cancer resistance protein (BCRP, also called ABCG2) produces MDR in a broad range of human cancers (13,14). ABCG2, a 72 kDa protein, is known as a half transporter that effluxes or extrudes molecules with amphiphilic characteristics (15). The substrates of ABCG2 include sulfated hormone metabolites, methotrexate, mitoxantrone (MX), topotecan and irinotecan (16). ABCG2 is a widely distributed transporter that is present mainly in the plasma membrane, and is highly expressed in the placental syncytiotrophoblasts, apical surface of small intestines, colon epithelium, liver canalicular membrane, luminal surfaces of microvessel endothelium of human brain and in the veins and capillaries of blood vessels (17–20). Its wide distribution and expression suggests that it is involved in protecting the fetus and adult against endogenous and exogenous toxins (21). ABCG2 is also abundantly expressed in the placenta and is also called ABCP1 (ABC transporter expressed in placenta) (22). It is expressed in colon cancer cells resistant to MX, thereby giving ABCG2 the name MX resistant protein (MXR) (23). Mutations in the ABCG2 gene produce distinct substrate preferences within the mutant and wild-type variants. For example, a mutation at position 482 is the most important mutation for the determination of substrate specificity (24). The amino acid arginine (Arg or R) is located on the carboxy terminal of the third transmembrane segment of the membrane spanning domain, where substrate binding occurs probably due to the formation of salt bridges (15). These mutations cause conformational changes and alter the drug binding and efflux capacity of the transporter (25–27). The replacement of Arg with threonine (Thr or T) or glycine (Gly or G) at position 482 produces changes in the substrate profiles among the variants (15,28). Indeed, the multidrug efflux pump ABCG2 has been implicated as the cause of the ‘side population’ which helps define adult stem cells of various tissues and tumors, including placental trophoblasts, neural stem cells or progenitors and hematopoietic progenitors (29,30).

Numerous studies over the past 3 decades have shown that MDR in cancer cells can be attenuated or even reversed by the inhibitors of ABC transporters (26,31–34). However, many of these ABC transporter inhibitors, at concentration that reversed MDR, also produced unacceptable toxicity as well as problematic pharmacokinetic interactions (35). These limitations prompted the development of a number of new compounds that are more potent and selective. Recently, we have reported that several tyrosine kinase inhibitors (TKIs), including tivozanib (36), imatinib (37), nilotinib (38), lapatinib (32) and erlotinib (39), can reverse ABC transporter mediated MDR. However, none of these MDR inhibitors have been used clinically in combination with conventional anti-neoplastic drugs.

Masitinib, a novel phenyl aminothiazole derivative, is a TKI used in the management of various diseases including multiple sclerosis (40,41), asthma (42,43), rheumatoid arthritis (44,45) and neoplasmic conditions such as gastro-intestinal stromal tumor, and pancreatic cancer (46–49). In a phase II trial, masitinib significantly increased the overall survival rate and progression-free survival in patients with locally advanced or metastatic gastro-intestinal stromal tumor (48). Masitinib, in combination with gemcitabine, significantly increased the media time-to-progression in patients with advanced pancreatic cancer compared to gemcitabine-treated patients (48,49). Currently, no studies have examined the effect of masitinib on ABCG2-mediated MDR. Therefore, in this study, we examined the effect of masitinib on MDR to various antineoplastic drugs in HEK293 and H460 cells overexpressing ABCG2 (14,50,51).

## Materials and methods

### Reagents

[^3^H]-MX (4 Ci/mmol) was purchased from Moravek Biochemicals, Inc. (Brea, CA) Dulbecco’s modified Eagle’s medium (DMEM), fetal bovine serum (FBS), penicillin/streptomycin and trypsin 0.25% were purchased from HyClone (Waltham, MA). A monoclonal antibody against GAPDH was purchased from Cell Signaling Technologies (Beverly, MA). The antibody BXP-21 against ABCG2 was obtained from Santa Cruz Biotechnology, Inc. (Santa Cruz, CA). Masitinib was purchased from LC Laboratories (Woburn, MA). MX, SN38 and cisplatin were purchased from Tocris Bioscience (Ellisville, MO). 3-(4,5-Dimethylthiazol-yl)-2,5-diphenyltetrazolium bromide (MTT), dimethyl sulfoxide (DMSO) and doxorubicin were obtained from Sigma-Aldrich Chemical Co. (St. Louis, MO). Nilotinib was obtained from Selleck Chemicals (Houston, TX).

### Cell lines

The HEK293/pcDNA3.1 (empty vector), wild-type HEK293/ABCG2-482-R2, mutant HEK293/ABCG2-482-G2 and mutant HEK293/ABCG2-482-T7 cells were established by transfecting HEK293 with either the pcDNA3.1 or vectors containing the full length ABCG2 containing either arginine (R), glycine (G), or threonine (T) at amino acid 482, respectively. The cells were cultured in a medium containing 2 mg/ml of G418 (52). The parental human non-small cell lung cancer H460 cells were grown in DMEM, supplemented with 5% heat-inactivated FBS. Resistant H460/MX20 cells were cultured in the above-mentioned medium with the addition of 20 nM MX. All the above cell lines were kindly provided by Dr Susan E. Bates and Dr Robert W. Robey (NCI, NIH, Bethesda, MD).

### Cell sensitivity by tetrazolium dye assay

A modified 3-(4,5-dimethylthiazol-2-yl)-2,5-diphenyltetrazolium bromide (MTT) assay was performed to detect the viability of the cells to anticancer drugs *in vitro* (53). The cell numbers seeded into the 96-well plates were 5,000/well for HEK293/pcDNA3.1, HEK293/ABCG2-482-R2, HEK293/ABCG2-482-T7 and HEK293/ABCG2-482-G2 cells and 6,000/well for H460 and H460/MX20. The MTT assay was run in triplicate and the compounds tested included MX (0.001 to 1 *μ*M), SN38 (0.001 to 1 *μ*M), doxorubicin (0.003 to 3 *μ*M), cisplatin (0.1 to 100 *μ*M), masitinib (1.25 and 2.5 *μ*M), nilotinib (2.5 *μ*M). After seeding cells in 180 *μ*l of medium in 96-well plates and incubation for 24 h at 37°C, 20 *μ*l of the appropriate anticancer drug at various concentrations was added (20 *μ*l of a fixed concentration of test compound for reversal experiments were added 1 h prior to adding the anticancer drugs). Subsequently, cells were incubated with anticancer drugs (in DMEM supplemented with 10% fetal bovine serum) at 37°C for 72 h. After 72 h, 20 *μ*l MTT (4 mg/ml) was added to each well. The plates were incubated at 37°C for another 4 h. The MTT/medium was removed from each well, and 100 *μ*l of DMSO was added to each well. The absorbance was read at 570 nm using an Opsys microplate reader (Dynex Technologies, Chantilly, VA). The degree of resistance was calculated by dividing the IC_50_ for the MDR cells by that of the parental sensitive cells. The degree of the reversal of MDR was calculated by dividing the IC_50_ for cells with the anticancer drug in the absence of masitinib or other reversal compounds by that obtained in the presence of masitinib or other reversal compounds.

### [^3^H]-MX accumulation assay

The HEK293/pcDNA3.1, HEK293/ABCG2-482-R2, HEK293/ABCG2-482-T7, HEK293/ABCG2-482-G2, H460 and H460/MX20 cell lines were harvested at 80% confluency in T75 flasks for this experiment. All cell lines were trypsinized with 0.25% trypsin after observing their confluency in T75 flasks under a microscope and cell count was done using a hemocytometer. Approximately 6×10^6^ cells were incubated at 37°C in DMEM supplemented with 10% FBS with and without masitinib concentrations of 1.25 and 2.5 *μ*M for 2 h. Subsequently, HEK293/pcDNA3.1, HEK293/ABCG2-482-R2, HEK293/ABCG2-482-T7, HEK293/ABCG2-482-G2, H460 and H460/MX20 cells were incubated with 0.01 *μ*M [^3^H]-MX for 2 h. Following incubation, the medium was removed and the cells were rinsed three times with cold phosphate buffer saline (PBS). The cells were lysed by adding 200 *μ*l of lysis buffer and transferred to scintillation vials. Each sample was placed in scintillation fluid and radioactivity was measured in a Packard TriCarb^®^ 1900CA liquid scintillation analyzer from Packard Instrument Company, Inc (Downers Grove, IL).

### [^3^H]-MX efflux assay

To measure [^3^H]-MX efflux, cells were prepared using the procedure discussed for the drug accumulation experiment and then incubated in fresh medium at 37°C at various times (0, 30, 60 and 120 min) in the presence or absence of the test compounds. After washing three times with ice-cold PBS, the cells were lysed by adding 200 *μ*l lysis buffer and transferred to scintillation vials. Each sample was placed in scintillation fluid and radioactivity was measured using a Packard TriCarb 1900CA liquid scintillation analyzer from Packard Instrument Company, Inc.

### Preparation of cell lysates

Approximately 6×10^5^ cells were harvested and suspended in PBS, followed by centrifugation at 2,000 rpm for 2 min, and the cells were washed twice with the PBS. Lysate buffer and 1% aprotinin were added to the suspension followed by vortexing. The resuspended cells were kept on ice for 30 min followed by centrifugation at 12,000 rpm for 20 min. The supernatant was separated and was stored at −80°C for the experiment. Protein concentrations in the vesicles were determined using the bicinchonic acid (BCA^TM^) based protein assay (Thermo Scientific, Rockford, IL).

### Immunoblot analysis

Equal amounts of total cell lysates (40 *μ*g protein) were resolved by sodium dodecyl sulfate polycrylamide gel electrophoresis and electrophoretically transferred onto polyvinylidene fluoride (PVDF) membranes. After incubation in a blocking solution (5% skim milk) of TBST buffer (10 mM Tris-HCl, pH 8.0, 150 mM NaCl, and 0.1% Tween-20) for 1 h at room temperature, the membranes were immunoblotted overnight with primary monoclonal antibodies against ABCG2 at 1:200 dilution or GAPDH at 1:1,000 at 4°C, and were then incubated for 3 h at room temperature with horseradish peroxide (HRP)-conjugated secondary antibody (1:1,000 dilution). The protein-antibody complex was detected by enhanced chemiluminescence detection system (Amersham, Piscataway, NJ). The protein expression was quantified by Scion Image Software (Scion Corp., Frederick, MD).

### Molecular modeling of ABCG2

The structure of masitinib was built using the fragment dictionary of Maestro v9.0. The energy was minimized by a Macromodel program v9.7 (Schrödinger, Inc., New York, NY) using the OPLSAA force field with the steepest descent followed by a truncated Newton conjugate gradient protocol. The low-energy 3D structures of masitinib were generated by LigPrep v2.3 and the parameters were defined based on different protonation states at physiological pH ± 2.0, and all possible tautomers and ring conformations. The ligand structures obtained from the LigPrep v2.3 run were further used for generating 100 ligand conformations for each protonated structure using the default parameters of mixed torsional/low-mode sampling function. The conformations were filtered with a maximum relative energy difference of 5 kcal/mol to exclude redundant conformers. The output conformational search (Csearch) file containing 100 unique conformers of masitinib were used as input for docking simulations into each binding site of the human ABCG2 transporter.

A homology model of ABCG2 was built using the mouse apoprotein (PDB ID: 3G5U) as a template (54). To identify drug binding sites on ABCG2 homology model, we generated various grids based on the following residues as centroids, for example, Arg482 (grid 1), Asn629 (grid 2), Arg383 (grid 3) and Leu241 along with Gly83 (grid 4). The choice of these residues was based on their involvement in ABCG2 function as determined through mutational experiments (52,55). The grid 2 generated using Asn629 as the centroid was found to have the best docking score; hence, docking discussion was based on binding mode of masitinib at this site. Glide v5.0 docking protocol was followed with the default functions (Schrödinger, Inc.). The top scoring masitinib conformation at Asn629 site of ABCG2 was used for graphical analysis. All computations were carried out on a Dell Precision 470n dual processor with the Linus OS (Red Hat Enterprise WS 4.0) (56).

### Statistical analysis

Differences of the parameters between two cell groups were analyzed by two tailed Student’s unpaired t-test. The *a priori* significance level was set at p<0.05.

## Results

### Masitinib significantly enhances the sensitivity of cells over-expressing ABCG2 to antineoplastic drugs

**Figure 1.**
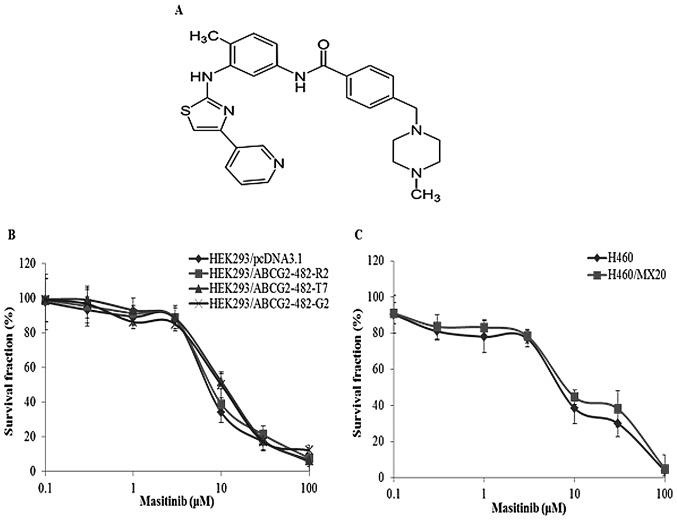
The chemical structure of masitinib and the effect of masitinib on the cell lines used in the study. (A) The chemical structure of masitinib (4-[(4-methylpiperazin-1-yl) methyl]-N-(4-methyl-3-{[4-(pyridin-3-yl)-1, 3-thiazol-2-yl] amino} phenyl) benzamide mesylate). (B) Cytotoxicity of masitinib in HEK293/pcDNA3.1, HEK293/ABCG2-482-R2, HEK293/ABCG2-482-T7 and HEK293/ABCG2-482-G2 cell lines. (C) Cytotoxicity of masitinib in H460 and H460/MX20 cell lines.

Cytotoxicity assays were performed to determine the non-toxic concentration of masitinib for the reversal studies (Fig. 1). It has been established that mutations at position 482 in ABCG2 can alter substrate and antagonist specificity of ABCG2 (15). Therefore, in the present study, both wild-type (R482) and two mutant forms (R482T and R482G) of ABCG2 were used. Masitinib, at 1.25 and 2.5 *μ*M, produced a concentration-dependent decrease in ABCG2-mediated resistance to MX, SN38 and doxorubicin as indicated by the decrease in the IC_50_ values (Table I). We also used nilotinib (2.5 *μ*M) as a positive control and as previously reported (38), it significantly decreased the resistance of wild-type HEK293/ABCG2-482-R2, mutant HEK293/ABCG2-482-T7 and mutant HEK293/ABCG2-482-G2 to MX, SN38 and doxorubicin respectively, as compared to HEK293/pcDNA3.1 cells (Table I). In addition, masitinib did not significantly alter the IC_50_ values for cisplatin, which is not a substrate of ABCG2 (Table I). These results suggest that masitinib enhances the sensitivity of ABCG2 substrates in both wild-type and R482T/G mutant ABCG2 overexpressing cells, i.e. it selectively reverses MDR.

Masitinib reversed ABCG2-mediated resistance to MX in transfected cell lines. Consequently, we determined whether masitinib could also reverse MX resistance in an ABCG2 overexpressing H460/MX20 lung cancer cell line that specifically confers resistance to MX. Masitinib, at 1.25 and 2.5 *μ*M, in combination with MX, SN38 or doxorubicin, significantly decreased the resistance of H460/MX20 cell line as compared to the parental H460 cell line. Nilotinib, 2.5 *μ*M, was used as a positive control and the results showed that it significantly decreased the resistance of H460/MX20 cells compared to that of controls. However, neither nilotinib nor masitinib (2.5 *μ*M) significantly altered the IC_50_ value of cisplatin (which is not an ABCG2 substrate) in H460 and H460/MX20 cells (Table II).

### Masitinib significantly increases the intracellular accumulation of [^3^H]-MX

**Figure 2.**
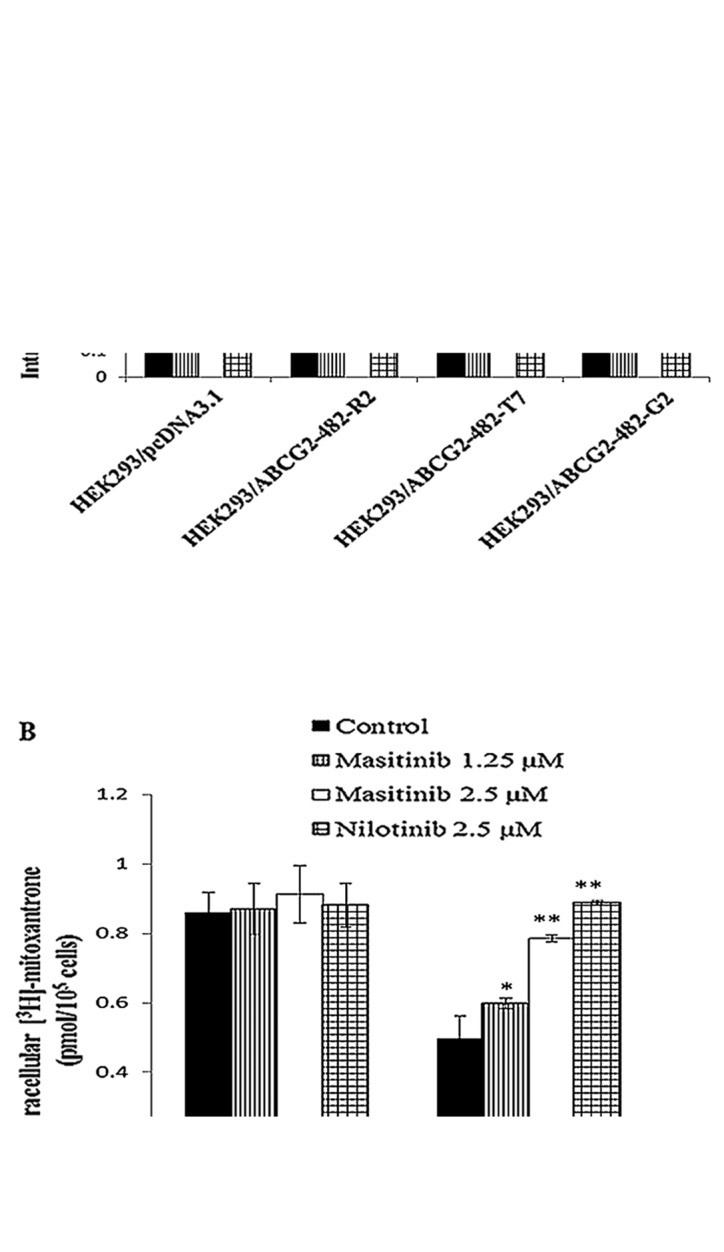
The effect of masitinib on the accumulation of [^3^H]-MX. The accumulation of [^3^H]-MX was increased in HEK293/ABCG2-482-R2, HEK293/ABCG2-482-T7 and HEK293/ABCG2-482-G2 cell lines in the presence of masitinib; ^*^p<0.05, ^**^p<0.01 vs. the control group. Error bars represent the SD. Experiments were performed at least three independent times (A). The accumulation of [^3^H]-MX was increased in H460/MX20 cell line in the presence of masitinib; ^*^p<0.05, ^**^p<0.01 vs. the control group. Error bars represent the SD. The experiments were performed at least three independent times (B).

In order to determine the mechanism by which masitinib attenuates ABCG2-mediated MDR, we measured the effect of masitinib on the intracellular accumulation of [^3^H]-MX, a known substrate of ABCG2. The incubation of wild-type HEK293/ABCG2-482-R2, mutant HEK293/ABCG2-482-T7, mutant HEK293/ABCG2-482-G2, and H460/MX20 cells with 1.25 or 2.5 *μ*M masitinib significantly increased the intracellular accumulation [^3^H]-MX in a concentration-dependent manner as compared to the parental HEK293/pcDNA3.1 and H460 cell lines (Fig. 2). Nilotinib (2.5 *μ*M), an inhibitor of ABCG2 (38), also significantly increased the accumulation of [^3^H]-MX.

### Masitinib significantly decreases the cellular efflux of [^3^H]-MX

**Figure 3.**
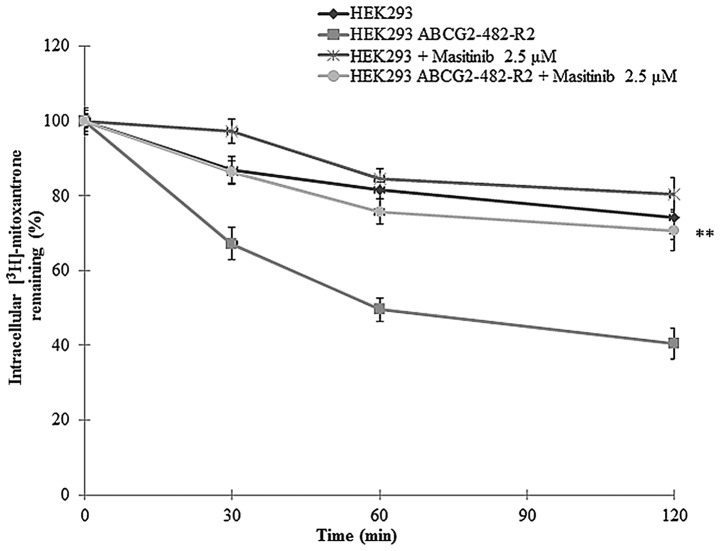
The effect of masitinib on the efflux of [^3^H]-MX. A time course vs. percentage of intracellular [^3^H]-MX remaining (%) was plotted (0, 30, 60, 120 min); ^**^p<0.01 vs. the control group. Error bars represent the SD. The experiments were performed at least three independent times.

In these experiments, we determined the amount of [^3^H]-MX present in the cells following incubation with masitinib. The amount of [^3^H]-MX present in the HEK293/ABCG2-482-R2 cell line was lower compared to HEK293/pcDNA3.1 cells due to the active efflux of [^3^H]-MX by the MDR transporter ABCG2. However, in the presence of masitinib (2.5 *μ*M), after 0, 30, 60 and 120 min the efflux of [^3^H]-MX was significantly reduced (Fig. 3).

### Masitinib does not alter the expression of ABCG2 protein levels

**Figure 4.**
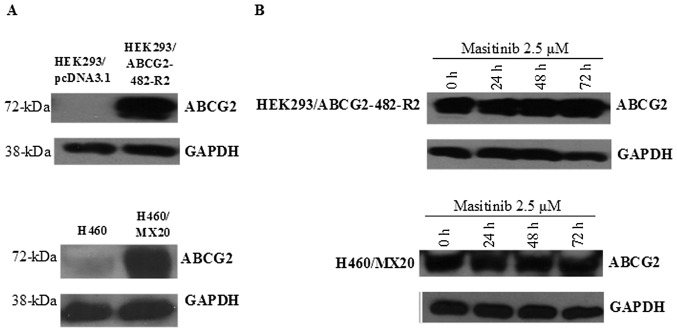
The effect of masitinib on the expression levels of ABCG2 transporter. (A) The expression of ABCG2 in HEK293/pcDNA3.1, HEK293/ABCG2-482-R2, H460 and H460/MX20 cell lysates. A representative result is shown and similar results were obtained in two other experiments. (B) The expression of ABCG2 protein in HEK293/ABCG2-482-R2 and H460/MX20 cells. A representative result is shown and similar results were obtained in two other experiments.

Immunoblot analysis indicated a band with a molecular weight of approximately 72-kDa in the HEK293/ABCG2-482-R2 and H460/MX20 cell lysates, suggesting the presence of the ABCG2 protein. However, this band was not present in the HEK293/pcDNA3.1 and H460 parental cell lines, indicating the absence of the ABCG2 protein in these cell lines (Fig. 4A).

In order to confirm that the masitinib-induced reversal of MDR was not due to a decrease in the expression of the ABCG2 protein, we measured the expression levels of ABCG2 in the cell lysates after incubation with masitinib (2.5 *μ*M) for 0, 24, 48 or 72 h. There was no significant change in the expression levels of the ABCG2 protein in HEK293/ABCG2-482-R2 and H460/MX20 cells (Fig. 4B). These findings suggest that the reversal of MDR by masitinib was not due to a decrease in ABCG2 protein expression.

### Model for binding of masitinib to ABCG2

**Figure 5.**
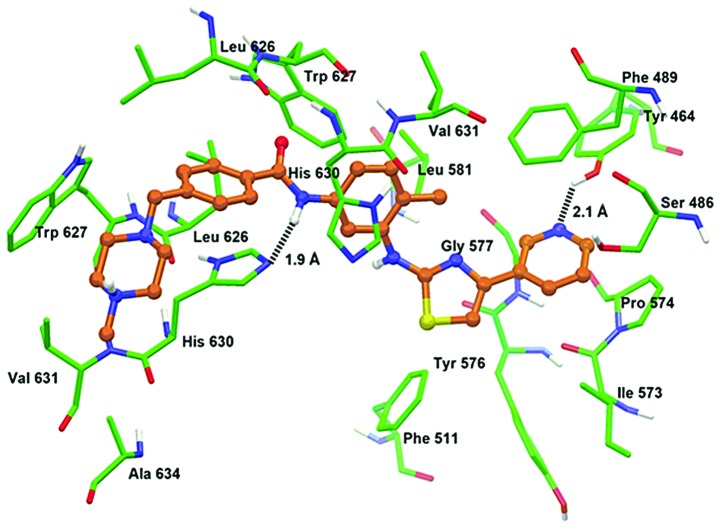
XP Glide predicted binding model of masitinib in the homology modeled ABCG2. The docked conformation of mastinib within the binding cavity of ABCG2 is shown as a ball and stick model. The important amino acids are depicted as sticks with the atoms colored as follows: carbon, green; hydrogen, white; nitrogen, blue; oxygen, red; whereas masitinib is shown with the same color scheme as above except the carbon atoms are presented in orange and sulfur atoms in yellow. The dotted black line indicates hydrogen-bonding interactions.

The XP-Glide predicted docked model of masitinib at Asn629 centroid-based grid of human ABCG2 is shown in Fig. 5. The (4-methyl-piperazin-1-yl) methyl-benzamide group formed hydrophobic interactions with the side chains of two copies of Leu626, Trp627 and His630 along with Val631 and Ala634. The amide group of the benzamide was involved in a hydrogen bonding interaction with the imidazole ring nitrogen of His630 (NH•••N-His630, 1.9 Å). The tolyl group was stabilized through hydrophobic contacts with the side chains of Phe489, Trp627, His630 and Val631. The thiazole ring and pyridine ring formed hydrophobic interactions with Tyr464, Phe489, Phe511, Ile573, Pro574, Tyr576 and Gly577. In addition, the pyridine ring nitrogen atom formed a hydrogen bond with the hydroxy group of Tyr464 (N•••HO-Tyr464, 2.1 Å). Docking studies were performed at various grid-based sites of human ABCG2 homology model.

## Discussion

One major finding of this study was that masitinib (1.25 and 2.5 *μ*M) significantly enhanced the sensitivity of HEK293/ABCG2-482-R2, HEK293/ABCG2-482-T7 and HEK293/ABCG2-482-G2 cells overexpressing the ABCG2 transporter to MX, SN38 and doxorubicin, which are substrates for the ABCG2 transporter (57–59). Specifically, masitinib produced a significant decrease in the IC_50_ values of the substrate drugs for the ABCG2 transporter in the MTT assay. In contrast, masitinib did not significantly alter the IC_50_ values for the aforementioned substrate drugs in parental HEK293/pcDNA3.1 or in H460 cancer cells which do not overexpress ABCG2 transporter. Furthermore, masitinib did not alter the sensitivity of HEK293/ABCG2-482-R2, HEK293/ABCG2-482-T7 and HEK293/ABCG2-482-G2 cell lines to cisplatin, a drug that is not a substrate for the ABCG2 transporter. These results suggest that masitinib significantly reverses MDR mediated by the overexpression of the ABCG2 transporter.

In order to gain insight into the mechanism of action of masitinib, we also assessed the effect of masitinib on i) the intracellular accumulation of [^3^H]-MX and ii) the efflux of [^3^H]-MX in wild-type HEK293/ABCG2-482-R2, mutant HEK293/ABCG2-482-T7, mutant HEK293/ABCG2-482-G2 and H460/MX20 cells. Masitinib produced a concentration-dependent increase in the response to the substrate drugs in the aforementioned cell lines but not in parental HEK293/pcDNA3.1 and H460 cell lines.

Masitinib significantly reverses MDR mediated by the overexpression of the ABCG2 transporter. Previously, it has been reported that small TKIs, including lapatinib (32), gefitinib (60), sunitinib (61) and apatinib (62) can reverse MDR in cell lines by inhibiting the efflux of the substrate drugs from the cells. In this *in vitro* study, masitinib (2.5 *μ*M) did not significantly alter the expression of the ABCG2 protein in HEK293/ABCG2-482-R2 or H460/MX20 cells. This suggests that reversal of MDR by masitinib is unlikely due to its decreasing the expression of the ABCG2 protein. This finding does not exclude the possibility that masitinib is preventing the translocation of the ABCG2 protein to the cell membrane (data not shown). To understand molecular interactions of masitinib, docking studies were performed at various grid-based sites of human ABCG2 homology model. According to its hydrophobic character (Clog P-value = 5.1), the inhibition of ABCG2 by masitinib may be explained by its significant distribution within the biomembrane, where it is extracted by the ABCG2 transporter. In addition, pharmacophoric features such as hydrophobic groups and/or aromatic ring center (phenyl ring, piperazine ring, pyridine ring and thiazole ring), hydrogen bond donor (-NH-) and hydrogen bond acceptor (pyridine nitrogen) have been reported critical for ABCG2 inhibition (63).

Our finding that masitinib reverses MDR and resensitizes cells by inhibiting the activity of the ABCG2 transporter may have clinical application. For example, there is a significant positive correlation between the overexpression of the ABCG2 transporter and MDR in many types of cells, including non-small cell lung cancer cells, thyroid and breast cancer cells as well as hematological malignancies (64–71). The presence of ABCG2 in esophageal squamous cell carcinoma and advanced non-small cell lung cancer is significantly correlated with a decreased survival (72–74). The ABCG2 transporter is present in certain populations (side population phenotype) of cancer stem cells and normal primitive stem cells and its presence increases the likelihood of resistance to various antineoplastic drugs (64,66,75–79). Overall, it is possible that masitinib, in combination with antineoplastics that are ABCG2 substrates, may be used in the treatment of certain MDR cancers. The validation of this hypothesis will require testing masitinib in clinical trials.

Collectively, the results of this *in vitro* study indicated that masitinib, at concentrations that were non-toxic to HEK293/pcDNA3.1 and H460 cells, significantly increased the toxic effects of substrate antineoplastic drugs in cells that overexpressed the ABCG2 transporter. This effect was most likely due to the masitinib inhibition of the efflux activity of the efflux transporter. Masitinib also reversed MDR in H460/MX20 lung cancer cells as indicated by their resensitization to MX, SN38 or doxorubicin. Our current results, provided they can be clinically translated, suggest that masitinib, in combination with other antineoplastics, may be efficacious in treating MDR cancers due to the overexpression of ABCG2 transporter.

## Figures and Tables

**Table I.** The effect of masitinib and nilotinib on the survival of HEK293/pcDNA3.1, HEK293/ABCG2-482-R2, HEK293/ABCG2-482-T7 and HEK293/ABCG2-482-G2 cells in the presence of MX, SN38, doxorubicin and cisplatin.

	HEK293/pcDNA3.1		HEK293/ABCG2-482-R2		HEK293/ABCG2-482-T7		HEK293/ABCG2-482-G2	
			
Compounds	IC_50_±SD^a^ (nM)	FR^b^	IC_50_±SD (nM)	FR	IC_50_±SD (nM)	FR	IC_50_±SD (nM)	FR
MX	20.39±2.1	1.0	172.82±6.6	8.5	534.38±18.9	26.2	588.62±28.8	28.8
+Masitinib 1.25 *μ*M	19.68±2.3	0.9	114.75±6.3^d^	5.6	332.19±38.7^d^	16.3	401.06±11.8^d^	19.6
+Masitinib 2.5 *μ*M	18.24±3.0	0.9	37.94±2.5^d^	1.9	45.15±1.8^d^	2.2	44.59±3.6^d^	2.2
+Nilotinib 2.5 *μ*M	16.24±3.6	0.8	18.5±1.5^d^	0.9	26.68±2.9^d^	1.3	28.46±1.5^d^	1.4
SN38	3.25±0.3	1.0	39.13±1.0	12.0	79.47±3.9	24.4	98.0±5.2	30.1
+Masitinib 1.25 *μ*M	2.59±0.1^c^	0.8	12.13±1.8^d^	3.7	69.4±0.7^d^	21.3	73.14±9.4^d^	22.4
+Masitinib 2.5 *μ*M	2.31±0.2^c^	0.7	3.54±0.6^d^	1.1	10.77±1.6^d^	3.3	12.16±2.6^d^	3.7
+Nilotinib 2.5 *μ*M	1.98±0.2^c^	0.6	2.25±0.5^d^	0.7	3.14±0.2^d^	0.9	6.07±0.6^d^	1.9
Doxorubicin	25.28±3.2	1.0	139.51±1.5	5.5	212.58±29.5	8.4	306.96±12.3	12.1
+Masitinib 1.25 *μ*M	23.75±2.6	0.9	90.35±4.7^d^	3.6	115.2±11.3^d^	4.5	266.07±9.7^d^	10.5
+Masitinib 2.5 *μ*M	21.41±2.0	0.8	41.43±2.8^d^	1.6	61.18±4.4^d^	2.4	84.23±10.2^d^	3.3
+Nilotinib 2.5 *μ*M	21.09±3.0	0.8	22.16±3.2^d^	0.9	34.91±6.7^d^	1.4	65.72±16.8^d^	2.5
Cisplatin	2,813.9±102.6	1.0	2,339.1±98.3	0.8	1,895.0±487.1	0.7	1,925.5±128.1	0.7
+Masitinib 2.5 *μ*M	2,778.7±231.0	1.0	2,265.7±84.1	0.8	2,052.0±224.8	0.7	1,958.7±240.7	0.7
+Nilotinib 2.5 *μ*M	2,836.4±74.9	1.0	2,719.9±186.1	0.9	2,542.81±294.9	0.9	2,600.4±353.0	0.9

a IC_50_, concentration that inhibited cell survival by 50% (mean±SD).

b FR, fold-resistance was determined by dividing the IC_50_ values of substrate in HEK293/ABCG2-482-R2, HEK293/ABCG2-482-T7 and HEK293/ABCG2-482-G2 cells by the IC_50_ of substrate in HEK293/pcDNA3.1 cells in the absence of masitinib; or the IC_50_ of substrate in HEK293/pcDNA3.1 cells in the presence of masitinib divided by the IC_50_ of substrate in HEK293/pcDNA3.1 cells in the absence of masitinib. Values in table are representative of at least three independent experiments performed in triplicate.

c p<0.05 or

d p<0.01, respectively, indicate statistically significant difference from IC_50_ of HEK293/pcDNA3.1, HEK293/ABCG2-482-R2, HEK293/ABCG2-482-T7, HEK293/ABCG2-482-G2 without reversal drug.

**Table II.** The effect of masitinib and nilotinib on the survival of H460 and H460/MX20 cells to MX, SN38, doxorubicin and cisplatin.

	H460		H460/MX20	
	
Compounds	IC_50_±SD^a^ (nM)	FR^b^	IC_50_±SD (nM)	FR
MX	41.91±3.0	1.0	3700.2±143.7	88.2
+Masitinib 1.25 *μ*M	33.45±2.1	0.8	203.1±9.3^c^	4.8
+Masitinib 2.5 *μ*M	31.0±0.5	0.7	61.47±2.2^c^	1.4
+Nilotinib 2.5 *μ*M	28.7±0.8	0.7	46.55±1.0^c^	1.1
SN38	20.77±1.4	1.0	1,414.7±191.5	68.0
+Masitinib 1.25 *μ*M	20.3±1.6	1.0	622.3±75.5^c^	30.0
+Masitinib 2.5 *μ*M	17.62±0.8	0.8	80.84±5.1^c^	3.9
+Nilotinib 2.5 *μ*M	15.74±1.1	0.7	39.61±3.3^c^	1.9
Doxorubicin	26.04±0.8	1.0	986.7±23.1	37.9
+Masitinib 1.25 *μ*M	25.08±1.5	1.0	218.2±11.2^c^	8.4
+Masitinib 2.5 *μ*M	25.43±1.1	1.0	52.61±1.2^c^	2.0
+Nilotinib 2.5 *μ*M	24.32±0.8	0.9	32.64±1.9^c^	1.2
Cisplatin	2,839.32±43.1	1.0	2,783.54±32.1	1.0
+Masitinib 2.5 *μ*M	2,532.54±35.2	0.9	2,343.23±12.3	1.2
+Nilotinib 2.5 *μ*M	2,742.55±23.1	1.0	2,711.98±53.7	1.0

a IC_5,_ concentration that inhibited cell survival by 50% (means±SD).

b FR, fold-resistance was determined by dividing the IC_50_ values of substrate in H460/MX20 cells by the IC_50_ of substrate in H460 cells in the absence of masitinib; or the IC_50_ of substrate in H460 cells in the presence of masitinib divided by the IC_50_ of substrate in H460 cells in the absence of masitinib. Values in table are representative of at least three independent experiments performed in triplicate.

c p<0.01 indicates statistically significant difference from IC_50_ of H460/MX20 without reversal drug.
